# Can HbA1c and One-Hour Glucose Concentration in Standard OGTT Be Used for Evaluation of Glucose Homeostasis in Childhood?

**DOI:** 10.4274/Jcrpe.889

**Published:** 2013-05-30

**Authors:** Gül Yeşiltepe Mutlu, Elif Özsu, Filiz Mine Çizmecioğlu, Şükrü Hatun

**Affiliations:** 1 Kocaeli University Medical Faculty, Department of Pediatrics, Division of Pediatric Endocrinology and Diabetes, Kocaeli, Turkey

**Keywords:** HbA1c, one-hour glucose concentration, impaired glucose tolerance, childhood

## Abstract

**Objective:** To investigate whether glycosylated hemoglobin (HbA1c) and 1-hour glucose level in oral glucose tolerance test (OGTT) are useful parameters for evaluation of glucose homeostasis in childhood.

**Methods:** The medical records of 106 obese/overweight children aged from 7 to 18 years who underwent OGTT were evaluated retrospectively. The subjects were divided into 2 groups according to their one-hour glucose concentration. Group 1 consisted of subjects whose one-hour glucose level was <155 mg/dL, and Group 2 consisted of subjects whose one-hour glucose level was ≥155mg/dL. The fasting and 2-hour glucose concentrations of the groups werecompared. The sensitivity and specificity levels were determined using the ROC curve to assess the predictive value of HbA1c for impaired glucose tolerance (IGT).

**Results:** The mean 2-hour glucose concentration of the subjects in Group 2 was significantly higher than that of the subjects in Group 1 (137.8±35.5 mg/dL versus 113.1±21.2 mg/dL, p<0.05). If a 5.5% cut-off value for HbA1c was accepted as predictor of IGT, the sensitivity was 63% and specificity was 70%. 31% of the subjects with HbA1c levels at or above 5.5% had IGT. This rate was significantly lower in subjects who had HbA1c levels below 5.5% (p<0.05).

**Conclusions:** Obese/overweight children and adolescents whose 1-hourglucose level is ≥155 mg/dL in the standard OGTT carry a high risk for IGT. Obese/overweight children and adolescents whose HbA1c level is at or above 5.5% may have IGT even though their fasting glucose level is normal, thus, OGTT is necessary to evaluate the glucose tolerance.

**Conflict of interest:**None declared.

## INTRODUCTION

In recent years, obesity has become more common in childhood and parallel to this trend, an increase in the prevalence of type 2 diabetes in childhood and adolescence has also occurred, creating a need for new parameters to evaluate glucose homeostasis in children (1). One-hour glucose concentration of ≥155 mg/dL in oral glucose tolerance test (OGTT) is considered as a strong marker for the development of diabetes in adults (2,3). Abdul-Ghani et al (2) suggested that 1-hour glucose concentration is more predictive of diabetes risk than the 2-hour glucose level in OGTT. Although the number of studies revealing this relationship in childhood is limited, Tfayli et al (4) showed the relationship between 1-hour glucose concentration and beta cell function regardless of glucose tolerance status in childhood.

Glycosylated hemoglobin (HbA1c) alone or in combination withother metabolic/clinicalparameters can be used as a screening methodfor detectingglucose intolerance in adults (5,6,7).

Studies in children and adolescents suggesting that HbA1c is more predictive of glucose intolerance than 2-hour glucose concentration in OGTT are limited (8,9). In this study, we therefore aimed to investigate whether HbA1c and 1-hour glucose in OGTT are useful parameters for evaluation of glucose homeostasis in children and adolescents.

## METHODS

The medical records of 106 obese/overweight children aged from 7 to 18 years (13.4±2.6, median: 13.5 years) who underwent OGTT between February 2010 and February 2011 were evaluated retrospectively. The subjects were divided into 2 groups according to their 1- hour glucose concentration. Children whose 1-hour glucose level was <155 mg/dL were included in Group 1, and Group 2 consisted of subjects whose 1-hour glucose level was ≥155 mg/dL. Body mass index (BMI), blood pressure, blood lipid profile, fasting glucose (FG), and 2-hour glucose concentrations of the groups were compared.

The subjects underwent a standard 2-hour OGTT, receiving an oral glucose load of 1.75 g/kg (maximum 75 g) after a 10-12-hour overnight fast. Blood samples were obtained at 0, 15, 30, 60, 90, and 120 min for determination of glucose and insulin levels. The lipid profiles (total cholesterol, triglyceride, HDL, LDL, VLDL cholesterol), liver transaminases (SGOT, SGPT) and HbA1c levels were measured in the 0 min samples. HbA1c levels were measured by high-performance liquid chromatography (HPLC). Biochemical values were measured by commercial enzymatic methods (Aeroset automated analyzer, Abbott Diagnostics, IL, USA).

BMI was calculated as weight (kg)/height (m)^2^, evaluated according to BMI reference percentiles of Turkish children, and was expressed as standard deviation score (SDS) (10). Obesity was defined as a BMI at or above the 95th percentile value and overweight as a BMI between 85th and 95th percentiles. Waist circumference (WC) was measured at the minimum circumference between the iliac crest and the rib cage. WC measurements were evaluated according to WC percentiles of Turkish children (11).

Impaired FG (IFG) was defined as a FG concentration between 100-125 mg/dL and impaired glucose tolerance (IGT) as a 2-hour glucose concentration between 140-199 mg/dL. Diabetes was defined as either a FG at or above 126 mg/dL or a 2-hour glucose concentration in OGTT at or above 200 mg/dL, as per the criteria of the American Diabetes Association (ADA) (12).

Homeostasis model assessment of insulin resistance (HOMA-IR) was calculated with the formula [(FG (nmol/L) x fasting insulin (mIU/mL)/22.5]. A level above 3.16 was accepted as a marker of insulin resistance (13).

The pubertal status of the subjects was evaluated according to Tanner staging, and stage 1 was accepted as prepubertal, stage 2-4 as midpubertal, and stage 5 as pubertal, respectively. 

## STATISTICAL ANALYSIS

The sensitivity and specificity levels were determined using ROC curve to assess the predictive values of HbA1c in subjects withIGT. We used Chi-square and t-tests for comparison of the groups, one-way ANOVA for the comparison of the means and Pearson method for correlation analysis, respectively.

## RESULTS

Mean BMI value of the subjects was 31.5±5.1 (20.7-46) kg/m^2^. Mean BMI-SDS was 2.6±0.6 SDS and mean WC was 94.6 cm. Forty-eight percent of the subjects were pubertal, 43% were midpubertal, and 9% were prepubertal.

Mean FG was 78.7±10 mg/dL (54-104 mg/dL), mean 2-hour glucose concentration was 119.6±27.8 mg/dL (50-238 mg/dL), and mean HbA1c level was 5.3±0.5% (4-7.5%). Three subjects (3%) had IFG, 18 subjects (17%) had IGT, and 1 subject (1%) had diabetes according to their 2-hour glucose concentrations. Only one of the 18 subjects who had an IFG had IGT. Mean 30-minute insulin concentration of the group was 102.3±83 uU/mL. Their mean plasma triglyceride level was 118.2±62.7 mg/dL, total cholesterol level 163.1±52.4 mg/dL, HDL cholesterol 43.5±11.8 mg/dL, LDL cholesterol level was 92.9±27.1 mg/dL, and VLDL cholesterol level 22.9±13.8 mg/dL (Table 1).

There was a negative correlation between the 2-hour glucose and the 30-minute insulin concentrations (p<0.01) and positive correlations between the 2-hour glucose concentration and the FG level and between the 1-hour glucose and the HbA1c levels (Table 2). However, the 2-hour glucose concentration was not correlated with age, pubertal stage, BMI, BMI-SDS, or WC (p=0.7, 0.6, 0.8, 0.9 and 0.7, respectively).

**HbA1c as a Predictor of IGT**

**Figure 1:**
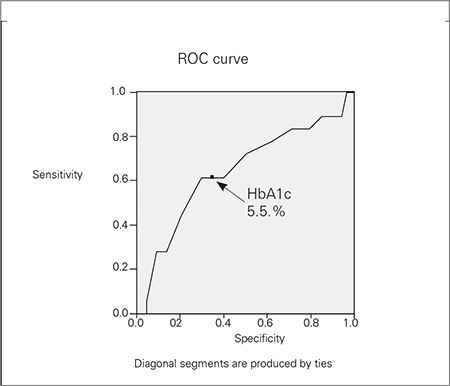
ROC curve

If a 5.5% cut-off value for HbA1c was accepted to be a predictor of IGT, the sensitivity was 63% and specificity was 70% (Figure 1). Although the cut-off values of 5.2 and 5.3% had higher sensitivity (78 and 72%, respectively), they had lower specificity (37 and 49%, respectively). 31% of the subjects who had HbA1c levels at or above 5.5% had IGT, however, this rate was significantly lower in the subjects who had HbA1c levels below 5.5% (10%) (p<0.05). Although only one (5.5%) of the 18 subjects with IGT had IFG, 12 (66.6%) of them had HbA1c at or above 5.5%.

**One-Hour Glucose Concentration as a Predictor of IGT**

When Group 1 (subjects whose 1-hour glucose concentration was below 155 mg/dL) and Group 2 (subjects whose 1-hour glucose concentration was ≥155 mg/dL) were compared in terms of BMI, WC, lipid profiles and HbA1c levels, no significant difference was observed. However, the mean values of FG and 2-hour glucose concentrations were significantly higher in Group 2 (Table 3).

## DISCUSSION

As in adults, obesity is a predisposing factor for type 2 diabetes in childhood and adolescence. The progression from normal glucose tolerance to type 2 diabetes mellitus involves an intermediate stage known as prediabetes or impaired glucose regulation, therefore the individuals who have IFG and/or IGT may be defined as prediabetic (14,15). Although OGTT is the gold standard for diagnosis of glucose intolerance and diabetes, quest for a simpler and inexpensive screening test has become a current issue due to the difficulties in the implementation of OGTT to large populations (16).

A number of studies about the usage of HbA1c alone or combined with clinical parameters as a screening test for diabetes in adulthood were published and different cut-off levels were suggested (6,7,17,18,19). In a study conducted on 2298 high-risk adults, for instance, when a 5.6% cut-off level for HbA1c was used to detect IGT, the specificity and sensitivity (51%, 66.2%, respectively) were lower than the combination of a ≥5.6 mmol/L cut-off level for FG and a ≥5.6% cut-off level for HbA1c (specificity was 82.4%, sensitivity was 87.9%) (6).

The number of studies suggesting HbA1c as a predictive parameter for IGT in childhood is limited (8,9,20). Tsay et al (9), who evaluated some parameters (FG, fasting insulin, triglyceride, cholesterol, blood pressure, BMI-Z score and HbA1c) as predictors of IGT, found that only HbA1c is a significant predictor of IGT. They suggested a HbA1c cut-off level of 5.5% (sensitivity 85.7%, specificity 56.9%) and detected a significant increase in 2-hour glucose concentration correlated with this HbA1c level (9). Shah et al (20) showed a moderate correlation between HbA1c, FG, HOMA-IR and 2-hour glucose concentration and reported that FG has the best correlation with 2-hour glucose concentration. In the same study, it was detected that a HbA1c cut-off level of ≥6% (specificity 96%, sensitivity 99%) is a good predictor of type 2 diabetes (19). In another adult study, the predictive value of FG in detecting IGT was shown to be more powerful compared to HbA1c. The predictive value of a cut-off level of 6.1 mmol/L for FG alone was reported to be satisfactory, and the use of combination of FG and HbA1c had no additional gain (21).

We found a significant correlation between 2-hour glucose levels and FG, HbA1c, and one-hour glucose levels. We suggest that the most appropriate cut-off level of HbA1c for predicting IGT is 5.5% (sensitivity63%, specificity 70%).

The frequency of IGT was 3 times higher among cases whose HbA1c levels were ≥5.5% as compared to cases whose HbA1c levels were <5.5%. IFG was detected in only 5.5% of the cases with IGT. However, HbA1c levels were at or above 5.5% in 66.6% of the cases with IGT. This relationship indicates that the obese/overweight children and adolescents with HbA1c levels of ≥5.5 are at high risk for IGT even though they do not have IFG.

We observed that 1-hour glucose concentration is correlated with 2-hour glucose concentration as well as with FG. Recent studies in adults have indicated the predictive value of 1-hour glucose concentration for the development of diabetes (2,3). In the San Antonio Heart Study, 1611 adults underwent OGTT. At the end of a 7-8 year follow-up period, the risk of type 2 diabetes was 2.9% among the cases whose 1-hour glucose concentration was <155 mg/dL, while the risk was higher (15.3%) in the cases whose 1-hour glucose concentration was >155 mg/dL (p<0.0001) (2). In another large study (the Botnia study), OGTT results in 2442 adults have shown that 1-hour glucose concentration was more powerful predictor for type 2 diabetes in comparison to 0-30-90-120-minute glucose concentrations (3).

Studies evaluating the predictive value of 1-hour glucose for development of type 2 diabetes in children and adolescent patients are scarce. Tfayli et al (4) found that obese/overweight children whose 1-hour glucose was ≥155 mg/dL had significant low beta cell function. Similarly, in our study, IGT frequency was higher among the cases whose 1-hour glucose was at or above 155 mg/dL. However, it is necessary to follow-up these cases for a longer period to evaluate the risk of type 2 diabetes.

In conclusion, the results of our study indicate that obese/overweight children and adolescents whose 1-hour glucose level is at or above 155 mg/dL in the standard OGTT have a high risk for IGT and subsequent type 2 diabetes. The obese/overweight children and adolescents whose HbA1c level is at or above 5.5% may have IGT even though their FG is normal, thus OGTT is necessary to evaluate glucose tolerance.

## Figures and Tables

**Table 1:**
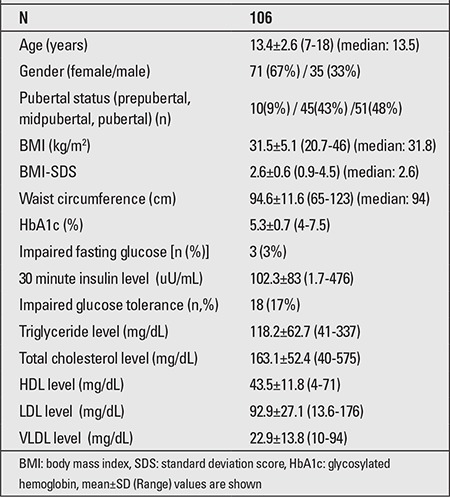
Demographic, clinical and laboratory values of the study group

**Table 2:**
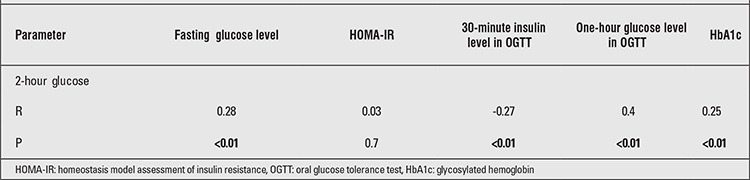
Correlations between 2-hour glucose concentrations and some parameters

**Table 3:**
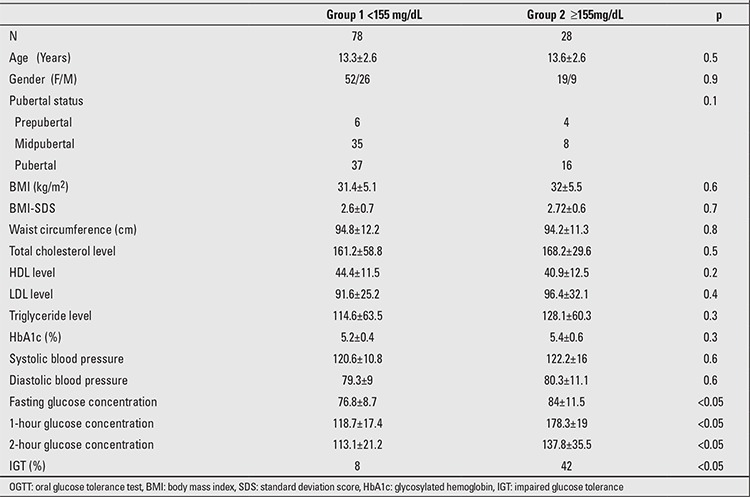
One-hour OGTT glucose levels in the two groups
